# Complement in Non-Antibody-Mediated Kidney Diseases

**DOI:** 10.3389/fmed.2017.00099

**Published:** 2017-07-12

**Authors:** Andrea Angeletti, Joselyn Reyes-Bahamonde, Paolo Cravedi, Kirk N. Campbell

**Affiliations:** ^1^Department of Medicine, Division of Nephrology, Icahn School of Medicine at Mount Sinai, New York, NY, United States; ^2^Department of Experimental Diagnostic and Specialty Medicine (DIMES), Nephrology, Dialysis and Renal Transplant Unit, St Orsola Hospital, University of Bologna, Bologna, Italy

**Keywords:** complement system, glomerular disease, thrombotic microangiopathy, fibrosis, focal segmental glomerulosclerosis

## Abstract

The complement system is part of the innate immune response that plays important roles in protecting the host from foreign pathogens. The complement components and relative fragment deposition have long been recognized to be strongly involved also in the pathogenesis of autoantibody-related kidney glomerulopathies, leading to direct glomerular injury and recruitment of infiltrating inflammation pathways. More recently, unregulated complement activation has been shown to be associated with progression of non-antibody-mediated kidney diseases, including focal segmental glomerulosclerosis, C3 glomerular disease, thrombotic microangiopathies, or general fibrosis generation in progressive chronic kidney diseases. Some of the specific mechanisms associated with complement activation in these diseases were recently clarified, showing a dominant role of alternative activation pathway. Over the last decade, a growing number of anticomplement agents have been developed, and some of them are being approved for clinical use or already in use. Therefore, anticomplement therapies represent a realistic choice of therapeutic approaches for complement-related diseases. Herein, we review the complement system activation, regulatory mechanisms, their involvement in non-antibody-mediated glomerular diseases, and the recent advances in complement-targeting agents as potential therapeutic strategies.

## Introduction

The complement cascade consists of 30 molecules that are activated as a proteolytic cascade regulated by three initiating pathways that function to protect the body from invading microorganisms (1, 2). Abnormal complement activation is also involved in many autoimmune inflammatory diseases. In particular, the pathogenesis of autoantibody-initiated kidney glomerulopathies suggests a role for complement-derived effector mechanisms leading to recruitment of infiltrating lymphocytes (3).

More recently, evidence has implicated a role for complement also in the pathogenesis of non-antibody-mediated kidney diseases that will be the topic of this review article. We will also discuss recent advances in complement-targeting strategies as potential therapeutic strategies for kidney disease.

### The Complement Cascade

#### Activation and Amplification

**Figure 1:**
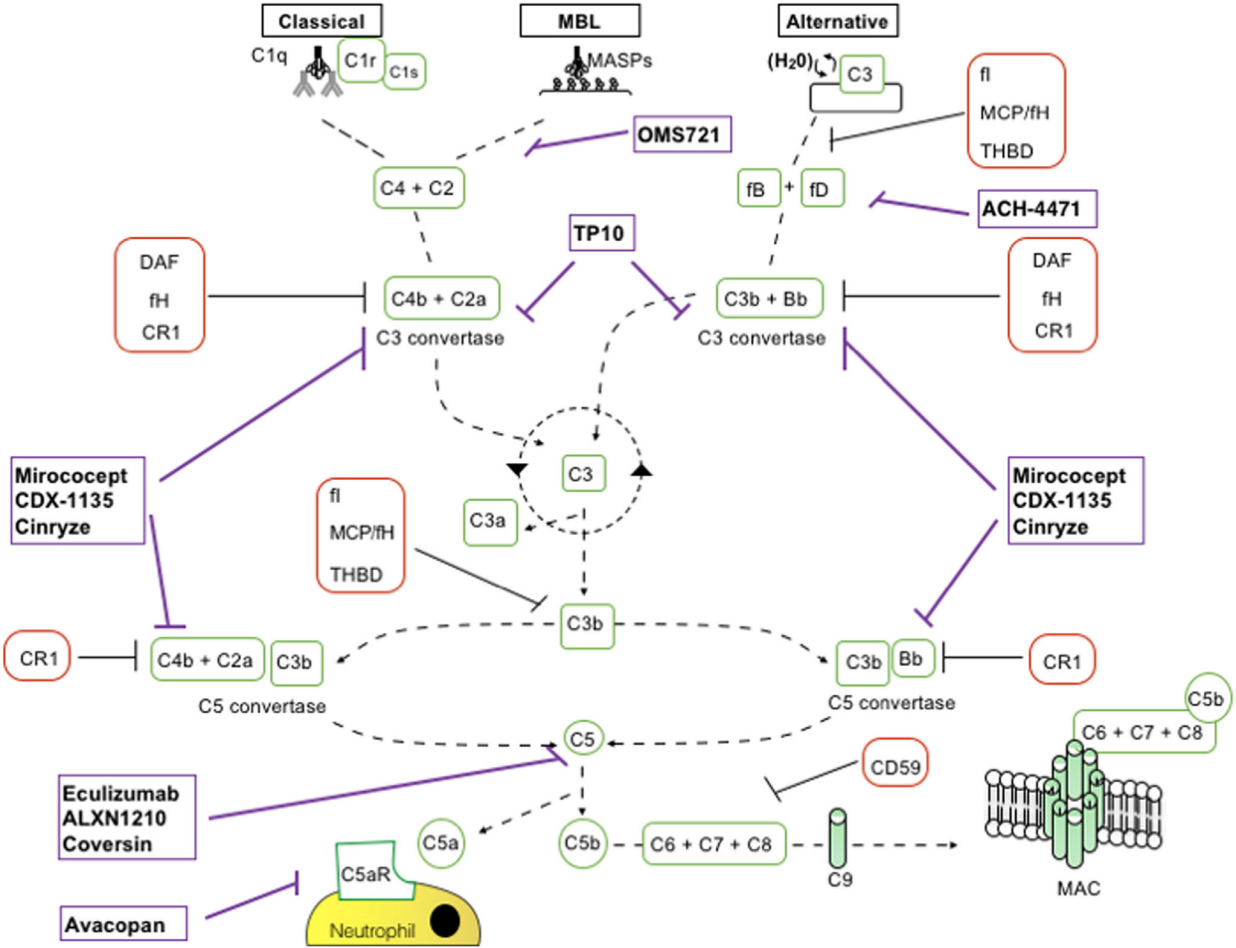
Schematic representation of complement activation pathways and complement-targeting agents. C1q,r,s cross-linking of antibodies activates the classical pathway. Mannose-associated serine proteases (MASPs) bind to mannose motifs expressed on bacteria to activate complement *via* the mannose-binding lectin (MBL) pathway. Subsequent cleavage and assembly of C2 and C4 proteins form the C3 convertase. The spontaneous hydrolysis of C3 on cell surfaces leads to the alternative pathway (AP): C3 convertase dependent on factor B (fB), factor D (fD), and properdin. The resultant C3 convertases can continuously cleave C3; however, after they are generated, the AP C3 convertase dominates in amplifying production of C3b (green looping arrow). C3 convertases cleave C3 into C3a and C3b. C3b permits the formation of C5 convertase. C3b has further roles in opsonization and immune complex clearance. C5b, in conjunction with C6–C9, allows formation of the membrane attack complex (MAC) and subsequent pathogen lysis. Decay accelerating factor (DAF) (CD55) and MCP (CD46) are cell surface-expressed complement regulators that accelerate the decay of all surface-assembled C3 convertases, thereby limiting amplification of the downstream cascade. MCP and factor H (fH) also have cofactor activity: in conjunction with soluble fI, they irreversibly cleave C3b to iC3b, thereby preventing reformation of the C3 convertase. CD59 inhibits formation of the MAC.

The complement cascade is activated by the lectin pathway (LP), the classical pathway (CP), and the alternative pathway (AP) (Figure 1). These three pathways converge on C3 convertases, enzymatic multimeric protein complexes (2). C3 cleavage produces C3a and C3b, the latter triggering formation of C5 convertase. C5 cleavage results in formation of the membrane attack complex (MAC, C5b-9). Along with MAC, soluble and surface-bound split products, including C3a, C3b, iC3b, C3dg, and C5a, play a role in the inflammatory response (4).

#### Regulation

It is essential to self-cell viability that complement activation is strictly controlled (4). Several molecules with discrete and synergistic roles regulate C3 convertase activity. Decay accelerating factor (DAF) encoded by the CD55 gene is a 70 kDa cell-surface regulator of the complement system. DAF inhibits C3 and C5 convertases thereby preventing downstream complement activation (5–8). Membrane cofactor protein encoded by CD46 is another inhibitory complement receptor with cofactor activity for C3b, C4b, and serum factor I inactivation (9). Crry is the murine homolog of human CD46 that also exhibits decay accelerating activity (10). Factor H (fH), a 155 kDa soluble glycoprotein exhibits both decay accelerating and cofactor activity to regulate the AP. Other complement cascade regulators include CD59 (protectin), the surface-expressed CR1 (11), and C1 inhibitor, a protease inhibitor of the serpin superfamily that inhibits the classical and LPs by binding and inactivating C1r, C1s, MASP-1, and MASP-2.

#### Complement Effector Mechanisms

Deposition of the MAC in the cell membranes of target cells results in the formation of transmembrane channels that promote cell lysis and death. In eukaryotic nucleated cells MAC insertion but can induce cellular activation (12) and/or promote tissue injury (13) but does not usually result in lysis.

Several complement cleavage products have distinct effector functions. For example, C3a and C5a promote vasodilation and chemokine release through their transmembrane-spanning G protein-coupled receptors. In addition, they regulate neutrophil and macrophage chemoattraction and contribute to T-cell and antigen-presenting cell (APC) activation, expansion, and survival (14–17).

## Complement and Adaptive Immunity

The complement system’s role in innate immunity has been well established since the 1960s. Recently, complement has been found to act as a link between innate and adaptive immunity. Complement depletion decreases antibody production (18) through antigen-bound C3dg binding to CR2 (CD21). This facilitates antigen presentation to B cells and lowers the threshold for B-cell activation (19).

There is also evidence that locally produced complement acts as a regulator of T-cell immunity. During T cell and APC interaction, there is upregulation and secretion of C3, fB, and fD, C5 production, and upregulation of surface expression of C3aR and C5aR (20, 21). Locally generated C3a and C5a bind to their respective receptors to act as autocrine and paracrine stimulators of T cells and the APCs (20, 21). Subsequent signaling through these GPCRs in T cells activates phosphoinositide-3-kinase-γ and induces phosphorylation of phosphokinase B (AKT) (21, 22), upregulating the pro-survival protein Bcl-2 and downregulating the proapoptotic molecule Fas. Together, these complement-dependent mechanisms enhance T-cell proliferation and diminish T-cell apoptotic injury (22).

Regulatory T cells (Tregs) are essential for maintenance of self tolerance (23) with recent evidence showing that complement also regulates Treg induction, function, and stability (16). Peripheral, murine, natural regulatory T cells (nTregs) express C3aR and C5aR and signaling through these receptors inhibits Treg function (15). Genetic and pharmacologic blockade of C3aR/C5aR signal transduction in nTreg cells augments their *in vitro* and *in vivo* suppressive activity. Genetic deficiency or pharmacologic blockade of C3aR/C5aR signaling augments murine-induced regulatory T cell (iTreg) generation, stabilizes Foxp3 expression, and resists iTreg conversion to IFN-γ/TNF-α-producing effector T cells (16, 24). Pharmacologic antagonists to human C3aR and C5aR also augment *in vitro* generation and stability of human iTreg from naïve precursors (16, 24). These findings are an extension of previously published data that co-engagement of the T-cell receptor and the complement regulator CD46 promote regulatory IL-10 production (25). In summary, there is a crucial role for complement in modulating the balance between pathogenic and protective adaptive T-cell responses.

## Source of Complement Components in Kidney Diseases

Complement deposition in the kidney in antibody-mediated glomerulonephritis was traditionally considered to derive from the circulating pool (mainly produced by the liver) (26). Subsequent studies have shown gene expression of complement in human kidneys (27) and the ability of resident cells (glomerular, tubular, epithelial, and mesangial cells) to synthesize several proteins of the complement cascade, such as C2, C3, C4, factor B (fB), and factor H (fH) (28–30). Sacks et al. (31) demonstrated *in vivo* the renal production of C3. Song et al. (32) described a higher expression of C2, C3, and C4 and C1q in the tubular cells of normal human kidneys than in glomerular cells. While the dominant role of renal versus systemic complement has been shown to mediate the ischemia–reperfusion injury, the role of circulating versus local complement in other physiological and pathological conditions has not yet been fully elucidated. Together, data support the conclusion that different inflammatory stimulation upregulate complement production in kidney tissue (11); the local effect of inflammatory stimuli on local complement regulators, as DAF, is still to clarify.

## Complement in Glomerular Diseases

### Focal Segmental Glomerulosclerosis (FSGS)

Focal segmental glomerulosclerosis is characterized by focal and segmental obliteration of glomerular capillary tufts with increased matrix (33). The incidence of FSGS has increased over the past decades and it is one of the leading causes of nephrotic syndrome in adults (34). Spontaneous remission is rare (<5%) and presence of persistent nephrotic syndrome portends a poor prognosis with 50% of patients progressing to end-stage renal disease (ESRD) 6–8 years after initial diagnosis (35).

While there are FSGS forms secondary to obesity, use of different drugs including lithium and anabolic steroid (36), primary cases historically have been attributed to a T cell disorder (possibly an imbalance between conventional and Tregs) resulting in the secretion of circulating factor(s) that increase glomerular permeability to plasma proteins (37). The identity of these permeability factor(s) is, however, still controversial (38). The origin of cells secreting the circulating factor(s) is also unclear, with new data pointing at neutrophils, monocyte/macrophages (39–41), and bone marrow immature myeloid cells (42). Efficacy of B cell depleting therapies in FSGS also implicates a role of B cell in disease pathogenesis (37).

Abnormal complement activation has also been implicated in the pathogenesis of the disease. Lenderink et al. (43) showed that fB-deficient mice have lower proteinuria than WT controls in the adriamycin-induced FSGS model, suggesting that activation of AP has a pathogenic role. Similarly, Turnberg et al. (44) reported lower proteinuria and less glomerular and tubulointerstitial injury, in fD-deficient mice compared to WT.

In a model of FSGS due to protein overload (45), fH-deficient mice display higher C3b glomerular deposition and more severe lesions than WT controls. *In vitro* experiments indicate that C3a activates podocytes to release glial cell line-derived neurotrophic factor that mediates the recruitment of parietal epithelial cell (PEC) and formation of sclerotic lesions. Signs of PEC activation were observed in renal biopsies from 10 patients with FSGS (45) (Table 1).

**Table 1:** Complement involvement in non-antibody renal disease.

Focal segmental glomerulosclerosis	AP	C3, filtered through endothelium and glomerular basement membrane, activates through the AP and signals on podocytes to release glial cell line-derived neurotrophic factor that mediates the recruitment of parietal epithelial cell (PEC) in the glomerulus. PEC proliferation leads to sclerotic lesions
Membranoproliferative/C3 glomerulonephritis	AP	Mutations in complement components/regulators or acquired antibodies targeting complement components lead to excessive activation of the AP in the fluid phase, with glomerular deposition of complement debris
Atypical hemolytic uremic syndrome	AP	Environmental triggers may precipitate complement activation in subjects with genetic predisposition including mutations of complement components
Chronic kidney injury and fibrosis	MBL	Intrarenal complement activation, especially of C3, activates the renin–angiotensin system and the epithelial-to-mesenchymal transition
	AP	

*AP, alternative pathway, MBL, mannose-binding lectin*.

Intriguingly, locally produced complement is implicated in abnormal T cell activation observed in the anti-podocyte model of FSGS. DAF-deficient mice develop more severe histological and ultrastructural features of FSGS than WT or CD59-deficient mice and severity of FSGS is reduced by depleting CD4+ T cells from DAF-deficient mice (46). Furthermore, WT kidneys transplanted into DAF-deficient recipients developed FSGS, suggesting that renal DAF is not implicated in mediating disease in this model (46). Altogether, these data indicate a major role for systemic and local complement dysregulation in murine models of FSGS.

In humans with FSGS, glomerular deposition of IgM and C3 deposits is frequently detected (47). In the urine and plasma of patients with FSGS, activated fragments of the complement cascade, such as C3a, C3b, Ba, Bb, C4a, and sC5b-9, are increased compared to patients with other renal diseases such as antineutrophilic cytoplasmic antibody (ANCA) vasculitis and lupus nephritis or in healthy controls (48). These findings could reflect complement activation within the glomeruli, mesangium, and areas of sclerosis, while activated complement fragments in urine could be due to activation of filtered proteins within the tubular lumen or urinary collecting system. The presence of high levels sC5b-9 is consistent with the hypothesis that there is an activation of the AP in FSGS (48). Importantly, fBa levels in the urine had an inverse relation with estimated glomerular filtration rate, further supporting a pathogenic role in disease progression (48). However, more studies are needed to better dissect the involvement of the AP of complement in the pathogenesis of FSGS as well as the implication of other complement pathway in the pathogenesis of the disease. Results of these studies could provide the background for clinical trials testing the hypothesis that complement blockade improves outcomes of FSGS patients.

## Membranoproliferative/C3 Glomerulonephritis

C3 glomerular disease (C3GN) refers to a group of recently identified rare renal disorders characterized by the presence of C3 in the absence or in the presence of limited deposition of immunoglobulins in the renal tissue (49, 50). Clinical manifestations include proteinuria, hematuria, and approximately 50% of affected patients progress to kidney failure within 10 years from diagnosis (51). C3GN is subdivided into dense deposit disease (DDD) and C3 glomerulonephritis. DDD is characterized by mesangial and intramembranous highly electron-dense deposits, the composition of which has not been still completely clarify.

C3 glomerular disease shows isolated and less-dense deposits in the mesangial, subepithelial, subendothelial, and intramembranous areas of the glomeruli (52). Glomerular deposits of C3 alone, without immunoglobulin, are the hallmark of AP dysregulation *via* inherited or acquired defects. Reports from Servais et al. (53) showed frequently acquired or hereditary abnormalities mutation of the AP and MCP in about 24% of patients which included cases of C3GN, DDD, and membranoproliferative glomerulonephropathy type I (100% if only C3GN was included). This defect can be due to mutations in complement components or complement regulators (such as C3, fB, fH, and fI) or due to acquired autoantibodies that either stabilize the C3 convertase of the AP (e.g., C3 nephritic factors) or target the inhibitory complement factors (e.g., fH autoantibodies). In contrast to other diseases with AP involvement, these abnormalities promote excessive activation of the alternative complement pathway in the fluid phase, with deposition of complement debris, including breakdown products of C3b and components of the terminal complement cascade, in the glomerular capillary wall (54–56) (Table 1).

The optimal treatment for C3 glomerulopathy remains undefined. A recent KDIGO (Kidney Disease: Improving Global Outcomes) controversies conference recommended that all patients receive optimal blood pressure control and that patients with moderate disease (defined as urine protein of more than 500 mg/24 h despite supportive therapy or moderate inflammation on renal biopsy or recent rise in creatinine) receive prednisone or mycophenolate mofetil (57). Due to its pathogenesis, targeted therapies aimed at specific components of the alternative complement pathway may also be effective (58).

## Complement in Thrombotic Microangiopathies

Thrombotic microangiopathy (TMA) is characterized by the presence of thrombi in small blood vessels, thrombocytopenia, non-immune hemolytic anemia, and peripheral blood schistocytes. The two main target organs of TMA are the kidney and the brain (59). The common denominator in each TMA forms is activation and dysfunction of the endothelium (60). Multiple etiologies can lead to development of TMA, including infection with Shiga-like toxin-producing bacteria causing typical HUS (STEC-HUS), genetically determined dysregulation of the AP, which predisposes to atypical hemolytic uremic syndrome (aHUS), or A Disintegrin and Metalloproteinase with ThromboSpondin motif repeats 13 (ADAMTS13) deficiency resulting in thrombotic thrombocytopenic purpura (TTP). TMA may also develop as a complication of various coexisting diseases or their treatments, such as malignant hypertension, systemic autoimmune disease such as systemic lupus erythematosus, cancer, drug treatment, hematopoietic stem cell transplantation, solid organ transplantation, open heart surgery, glomerulopathies or infections (61).

In STEC-HUS, bacterial exotoxins induce profound alterations in endothelial cells, upregulating expression of chemokines, chemokine receptors, and cell adhesion molecules that favor leukocyte recruitment and promote platelets activity (62). From the early 1970s, there have been reports of low levels of C3 in patients with STEC-HUS (63–67). Since 1980, increased levels of breakdown products of the components of the AP including C3 convertase, C3, fB, and MAC have been reported in the plasma of children with STEC-HUS (68–70). These findings suggest the activation of the AP of complement cascade, through to C5b–9 assembly, in STEC-HUS. Low levels of C4 have also occasionally been observed indicating activation of the CP and/or LP leading to C4 consumption (70). Evidence shows that exotoxin might directly contribute to AP activation (71), through endothelial complement deposition and loss of thromboresistance depended on exotoxin-induced upregulation of the membrane adhesion molecule P-selectin, which has been shown to bind C3b with high affinity (72).

HUS is defined as atypical when the disease occurs in the absence of a STEC infection, according to established criteria (57). The onset of aHUS ranges from the neonatal period to adulthood. Genetic aHUS accounts for an estimated 60%of all aHUS (73). It is likely that mutation of *C3, CD46, CFB, CFH, CFI*, and *THBD* confers a predisposition to developing aHUS, rather than directly causing the disease. Conditions that trigger complement activation may precipitate an acute event in those with the predisposing genetic background (74, 75) (Table 1). There are, however, non-complement inherited abnormalities such as mutations in *DGKE*, which can result in an aHUS phenotype. Until recently, the prognosis for aHUS was poor, with the majority of patients developing ESRD within 2 years of presentation. However, with the introduction of eculizumab, a humanized monoclonal antibody against C5, it is now possible to control the renal disease and prevent development of ESRD (57).

In TTP, systemic platelet thrombi are mainly composed by platelets and von-Willebrand Factor (VWF) (76). VWF is a high-molecular weight, multimeric plasma glycoprotein, produced by endothelial cells with highly thrombogenic property mediated by the availability of an array of docking sites for platelets on endothelial cells and extracellular matrix collagen. In normal conditions, the VWF thrombogenic potential is rapidly held in check through cleavage into smaller multimers by a plasma metalloprotease, ADAMTS13 (77), thus ADAMTS13 deficiency predisposes to microvascular thrombosis after a triggering event. ADAMST13 activity could also be helpful in predicting the disease clinical manifestation or, a relapsing course (78). Several studies investigated complement activation markers in TTP patients with documented severe ADAMTS13 deficiency, showing lower serum levels of C3 and MAC during the acute phase (79, 80), correlating also with disease activity. No significant differences in levels of CP or LP activation markers were observed (80). The above data point toward complement activation, through to the terminal C5b–9 complex, in TTP, but how complement is activated in TTP is still unclear.

## Chronic Kidney Injury and Fibrosis

The pathogenesis of progressive renal fibrosis is complex and involves various cell types and molecular pathways. However, it is evident that the inflammatory microenvironment of the kidney, after sustained injury, plays a dominant role in the dynamic balance between tissue destruction (tubular atrophy and interstitial fibrosis) and repair (tubular cell growth and resolution of renal inflammation and fibrosis) (81). Recent evidence has implicated intrarenal complement activation in the progression of kidney injury of chronic renal failure (82). C3 plays a substantial role in the activation of the renin–angiotensin system and the epithelial-to-mesenchymal transition (83, 84). This is consistent with the concept that complement component generation by renal epithelial cells promotes tubular damage in proteinuria-associated renal disease. Further evidence is provided by the fact that absence/blockade of C5/C5aR1 (but not blocking MAC formation) limits kidney fibrosis in several animal models (85, 86). Taken together, these data suggest that kidney-derived complement participates in the progression of renal fibrosis.

In a murine model of ascending urinary tract infection C5aR1 deficiency or blockade not only reduces renal bacterial load at all stages of infection but also attenuates tissue inflammation and tubulointerstitial fibrosis, suggesting a pathogenic role for C5aR1 in experimental chronic kidney infection. Mechanistic studies suggest that C5aR1-mediated bacterial colonization of tubular epithelium, persistent local inflammatory responses, and impairment of the phagocytic function of monocytes/macrophages could contribute to the pathogenesis of chronic kidney infection (87) (Table 1).

## Therapy

The last decade has witnessed a growing interest from pharmaceutical companies in the development of complement inhibitors for the most disparate indications, largely supported by the exceptional results obtained with anti-C5 blocking antibody eculizumab (Table 2; Figure 1).

**Table 2:** Main complement-targeting therapies.

Name	Class	Disease	Main pathogenic mechanism	Status	Comments
Eculizumab	Humanized monoclonal anybody	aHUS DDD C3GN	Bind C5 to prevent generation of MAC	On the market	The first U.S. Food and Drug Administration approved anticomplement therapy
ALXN1210	Humanized monoclonal anybody	aHUS	Bind C5 to prevent generation of MAC	Clinical trial phase II	Demonstrated rapid, complete and sustained reduction go free C5 levels, requiring longer dosing intervals compared to eculizumab
Coversin	Recombinant small protein	aHUS	Prevents the cleavage of C5 into C5a and C5b by C5 convertase	Clinical trial phase I	Covers has the potential to treat patients with polymorphisms of the C5 molecule which interfere with correct binding of eculizumab
CCX168 (Avacopan)	Anti-inflammatory small molecule	aHUS ANCA vasculities	Selective inhibitor of the complement C5a receptor	Clinical trial phase III	C5a receptor inhibition with avacopan was effective in replacing high-dose glucocorticoids in treating vasculitis
CDX-1135	CR1-based protein	DDD	CR1 inhibitor	Clinical trial phase I	CDX-1135 has been shown safe in more than 500 patients in different clinical trials, with no relevant side effects
APT070 (Mirococept)	CR1-based protein	IRI in renal transplant	CR1 inhibitor	Clinical trial phase II	Mostly investigated in models of complement-mediated IRI, such as kidney transplantation in rats
					Phase I trial proved safety
Cinryze	C1 esterase inhibitor	Antibody-mediated rejection in renal transplant	C1 inhibitor	Clinical trial phase III	Efficacy will be tested proportion of subjects with new or worsening transplant glomerulopathy at 6 months using Banff criteria
OMS721	Humanized monoclonal anybody	aHUS TTP IgAN	Bind mannan-binding lectin-associated serine protease-2	Clinical trial phase II	OMS721 requests a multidose administration
ACH-4471	Small molecule	aHUS	Factor D inhibitors	Clinical trial phase I	ACH-4471 can be given orally and would have a delivery advantage over intravenously infused agents

*aHUS, atypical hemolytic uremic syndrome; DDD, dense deposit disease; C3GN, C3 glomerular disease; MAC, membrane attack complex; ANCA, antineutrophilic cytoplasmic antibody; IRI, ischemia–reperfusion injury; TTP, thrombotic thrombocytopenia; IgAN, IgA nephropathy*.

### Eculizumab

Eculizumab is the first complement inhibitor approved for clinical use, initially for paroxysmal nocturnal hemoglobinuria (PNH) and subsequently for aHUS (88). It is an anti-C5 humanized mAb which prevents C5 cleavage by the C5 convertase, thereby inhibiting the terminal complement effector pathway. In absence of C5b the assembly of MAC is prevented (88).

Eculizumab was approved by the European Medicines Agency’s and the US Food and Drug Administration for the treatment of aHUS on the basis of results from two distinct prospective trials in 17 aHUS patients with thrombocytopenia and in 20 aHUS patients requiring persistent plasma exchange (PE), respectively (89). All patients achieved discontinuation of PE and 88% (33 of 37) reached normal hematological values after median of 63 weeks of eculizumab treatment. Eculizumab has clearly improved the renal outcome of aHUS patients with a dramatic decrease in the risk of ESRD. Nevertheless, the clinical use of eculizumab in aHUS still carries a number of unanswered questions, mostly concerning patient selection, timing and duration of the treatment: an ongoing multicenter single-arm trial is testing the safety of the discontinuation of eculizumab treatment in patients with aHUS (NCT02574403).

Efficacy of eculizumab in patients with DDD and C3GN, limited to case reports, suggested that eculizumab is a promising option in native disease, but not in C3GN recurrence after kidney transplantation (90–98). The only open-label study of eculizumab therapy in six patients (three C3GN and three DDD) reported complete to partial remission in four patients at one year of follow-up (99, 100).

### Inhibitors of Terminal Effector Complement

Novel anti-C5 mAb antibodies are expected to reproduce the efficacy of eculizumab with longer half-lives and at lower costs. An ongoing single-arm study is testing the efficacy in controlling disease activity of a longer-acting anti-C5 humanized monoclonal antibody (ALXN1210) in patients with aHUS who have not previously used a complement inhibitor (NCT02949128).

Coversin is a recombinant small animal protein complement C5 inhibitor able to prevent the cleavage of C5 into C5a and C5b by C5 convertase (101). *In vitro*, it prevents hemolysis of PNH erythrocytes (102). In an open label, non-comparative clinical trial, Coversin reducing serum lactic dehydrogenase, will be tested in patients with PNH and proven resistance to eculizumab due to C5 polymorphisms (NCT02591862).

CCX168 (Avacopan) is a small molecule C5aR inhibitor. Jayne et al. recently conducted a randomized, double-blind, placebo-controlled trial in 67 adults with ANCA-associated vasculitis, treated with Avacopan with or without steroids. All patients received cyclophosphamide or rituximab. They achieved treatment responses in 86 and 81% of the avacopan with and without steroid groups, respectively, and in 70% in the control group, meeting non-inferiority criteria (103). One more randomized phase III trial Avacopan in patients with ANCA-associated vasculitis is in recruiting participants (NCT02994927), and an open-label phase II study to assess the effect of C5aR inhibitor in aHUS is in terminal status (NCT02464891).

TP10 is a C3 convertase inhibitor that acts as a soluble complement receptor. TP10 can be considered as a candidate for C3G and a phase I trial is currently underway (NCT02302755).

### Inhibitors of Initial Complement Activation

Acting at the level of initial pathway-specific events that lead to C3 activation could represent an effective therapy to prevent C3 activation. Several preclinical trials are testing this strategy (102), and it is particularly useful in diseases where a specific complement pathway has a dominant pathogenic role. Examples would be the AP in PNH or the CP in antibody-mediated hemolytic anemias.

CR1 is a regulator of complement activation inhibiting C3/C5 convertases with effect on all the three complement pathway (104, 105). A soluble form of CR1, named CDX-1135 has been shown strongly effective in mouse model of DDD (106), and some clinical benefit has been reported in a short-term compassionate therapy in a child (106). A phase I clinical trial for testing CDX-1135 in DDD has been started and terminated (NCT01791686) with no results reported.

APT070 (Mirococept) (107) is an engineered molecule consisting of the first three short consensus domains of CR1and it is able to link it to cell membranes. Mirococept has been investigated in IRI preclinical studies achieving a significant increase in the number of surviving grafts, compared with control-treated grafts (63.6 versus 26.3%) (108). An ongoing phase III randomized, placebo-controlled trial is testing Mirococept as a protective agent to prevent functional impairment of transplanted kidneys (http://www.controlled-trials.com/ISRCTN49958194). Cinryze, a C1 esterase inhibitor currently used for hereditary angioedema, is being evaluated in a randomized double-blind study for the treatment of acute antibody-mediated rejection in donor-sensitized kidney transplants recipients (NCT02547220).

An antibody (OMS721) targeting mannan-binding lectin-associated serine protease-2 that cleaves C4 and C2, is currently being tested in clinical trials for use in TMA, aHUS (NCT02222545), and IgA nephropathy (NCT02682407).

Factor D inhibitors are agents that mitigate the complement-mediated amplification step of the AP. ACH-4471, a small molecule, demonstrated the ability to block the activation of the AP achieving decrease hemolysis and C3b deposition on red blood cells from patients with PNH (109) ACH-4471 is being tested in phase 1 clinical trials (110).

## Conclusion

Increasing evidence has been accumulated showing that complement activation (mainly through the AP) is implicated in the pathogenesis of different non-antibody-mediated glomerular diseases and in the general progression of renal disease, regardless of the initial insult.

Eculizumab, an anti-C5 monoclonal antibody approved for the treatment of aHUS and PNH, revolutionized the treatment of TMA, with safe and effective inhibitors for different levels of complement cascade emerging. The advent of selective complement-targeting therapies has the potential provide new treatment options while enhancing our understanding of complement involvement in disease pathogenesis.

## Author Contributions

All the authors participated in the drafting and critical appraisal of the manuscript, approved the final version, and agreed to be accountable for all aspects of the work.

## Conflict of Interest Statement

The authors declare that the research was conducted in the absence of any commercial or financial relationships that could be construed as a potential conflict of interest.
